# Limitations of Ultrathin Al_2_O_3_ Coatings on LNMO Cathodes

**DOI:** 10.1021/acsomega.1c04457

**Published:** 2021-11-03

**Authors:** Elise R. Østli, Yonas Tesfamhret, Sigurd Wenner, Matthew J. Lacey, Daniel Brandell, Ann Mari Svensson, Sverre M. Selbach, Nils P. Wagner

**Affiliations:** † Department of Materials Science and Engineering, NTNU Norwegian University of Science and Technology, 7491 Trondheim, Norway; ‡ Department of Chemistry−Ångström Laboratory, Uppsala University, P.O. Box 538, 75121 Uppsala, Sweden; § Sintef Industry, 7491 Trondheim, Norway; ∥ Scania CV AB, 151 32 Södertälje, Sweden

## Abstract

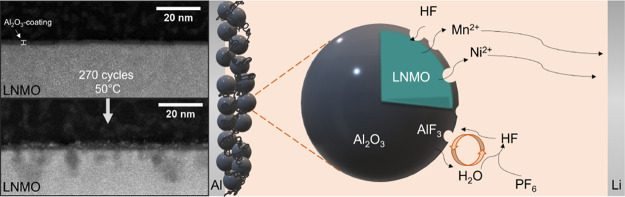

This study demonstrates the application of Al_2_O_3_ coatings for the high-voltage cathode material LiNi_0.5–*x*_Mn_1.5+*x*_O_4−δ_ (LNMO) by atomic layer deposition. The ultrathin and uniform coatings (0.6–1.7 nm) were deposited on LNMO particles and characterized by scanning transmission electron microscopy, inductively coupled plasma mass spectrometry, and X-ray photoelectron spectroscopy. Galvanostatic charge discharge cycling in half cells revealed, in contrast to many published studies, that even coatings of a thickness of 1 nm were detrimental to the cycling performance of LNMO. The complete coverage of the LNMO particles by the Al_2_O_3_ coating can form a Li-ion diffusion barrier, which leads to high overpotentials and reduced reversible capacity. Several reports on Al_2_O_3_-coated LNMO using alternative coating methods, which would lead to a less homogeneous coating, revealed the superior electrochemical properties of the Al_2_O_3_-coated LNMO, suggesting that complete coverage of the particles might in fact be a disadvantage. We show that transition metal ion dissolution during prolonged cycling at 50 °C is not hindered by the coating, resulting in Ni and Mn deposits on the Li counter electrode. The Al_2_O_3_-coated LNMO particles showed severe signs of pitting dissolution, which may be attributed to HF attack caused by side reactions between the electrolyte and the Al_2_O_3_ coating, which can lead to additional HF formation. The pitting dissolution was most severe for the thickest coating (1.7 nm). The uniform coating coverage may lead to non-uniform conduction paths for Li, where the active sites are more susceptible to HF attack. Few benefits of applications of very thin, uniform, and amorphous Al_2_O_3_ coatings could thus be verified, and the coating is not offering long-term protection from HF attack.

## Introduction

Energy storage and electrification of the transport sector are critical measures for reducing global greenhouse gas emissions. Secondary batteries have been shown to be a viable solution of energy storage for consumer electronics as well as electric propulsion and stationary energy storage. For the former two applications, batteries with high energy density are of utmost importance. The best-performing battery technology to date with respect to energy density and cycle life are secondary Li-ion batteries (LiBs) where the cathode consists of a Li transition metal (TM) oxide. The use of scarce resources such as Co and Ni in the cathode is a major cost factor and has a negative impact on the environmental footprint.^1^ As the fleet of electric vehicles is expected to grow immensely over the next years,^2^ alternative high-energy cathode materials based on abundant materials are in urgent need. The two main factors that determine the energy density of a battery are the specific capacity of the electrode materials and their difference in the electrochemical potential. Considerable effort has been put into the development of improved, high-capacity cathode materials such as nickel-rich layered oxides, where the reversible capacity is increased by increasing the Ni content.^3,4^ Another route to increase the energy density is the implementation of high-voltage cathode materials. With its notably high operating potential of 4.7 V versus Li/Li^+^, LiNi_0.5–*x*_Mn_1.5+*x*_O_4−δ_ (LNMO) stands out as a promising cathode material for use in the next-generation LiBs. LNMO yields a comparable energy density to nickel-rich LiNiMnCoO_2_ (NMC) cathodes, while the reduction in Ni content (0.16 g Ni/1 g LNMO vs 0.48 g Ni/1 g NMC811) and the absence of Co will reduce both the price and the environmental footprint of the former.^1,5^ Even if the high operating potential increases the risk of CO and CO_2_ evolution from electrolyte decomposition reactions, the onset potential of these reactions has been shown to be high for LNMO compared to NMC.^3,6^ Still, issues concerning the unstable cathode/electrolyte interface lead to both TM dissolution and severe electrolyte degradation.^7,8^ TM dissolution has been observed for several cathode materials and is thus not a problem only associated with the high operating voltage of LNMO.^9,10^ The Mn-ion dissolution in the spinel LiMn_2_O_4_ (LMO) has been assigned to the disproportionation reaction (2Mn^3+^ → Mn^2+^ + Mn^4+^) and Jahn–Teller distortions.^11−13^ While the Mn^3+^ content in LNMO is reduced compared to that in LMO due to the partial substitution of Mn^3+/4+^ by Ni^2+^, synthesis conditions that induce structural disorder, oxygen deficiencies, and rocksalt-structured impurity phases (such as Li_*x*_Ni_1–*x*_O^14^) can generate Mn^3+^ in LNMO.^15,16^ Disproportionation reactions and the resulting TM dissolution are thus a problem also for LNMO. The dissolved TM ions will migrate over to the anode and deposit, interfering with the insulating characteristic of the solid electrolyte interphase (SEI). Pieczonka et al.^7^ observed metallic Mn and Ni particles on the graphite electrode from a 100 cycle LNMO∥graphite full cell, indicating that the TM ions are reduced on the graphite surface. The following continuous formation of new SEI will consume the cyclable Li and be detrimental to the cycle life in full cells with a finite amount of cyclable Li.^17^

The commercial LiB electrolytes contain LiPF_6_ salt in carbonate solvents such as ethylene carbonate (EC), diethyl carbonate (DEC), dimethyl carbonate, and ethyl methyl carbonate.^18^ Several of the electrolyte components are not stable at the high operating voltage of LNMO. As an example, EC will polymerize on the LNMO surface to form a polyethylene carbonate film.^8^ Furthermore, LiPF_6_ hydrolyses readily with a trace amount of water forming HF and POF_3_.^7,19−21^ HF can in turn attack the LNMO material and lead to increased TM dissolution. These unfortunate effects are thus shortening the lifetime of the battery and must be resolved before LNMO-based LiBs can be fully commercialized.

Protective surface coatings have been suggested as a viable strategy to protect the LNMO surface from HF attack and prevent TM dissolution.^22−24^ The chemically simple, cheap, and abundant Al_2_O_3_ has been widely investigated as a possible protective surface coating for electrodes in LiBs.^25^ The relatively low ionic and electronic conductivity of most Al_2_O_3_ phases,^26^ however, makes the coating thickness critical, in particular when amorphous coatings are applied. Ultrathin coatings are therefore desirable. Both Song et al.^27^ and Park et al.^28^ reported improved capacity retention for ultrathin atomic layer deposition (ALD) coatings of composite electrodes containing in-house synthesized LNMO. Coating of the entire electrode laminate will, in addition to protecting the active material, also protect the carbon black additive from direct contact with the electrolyte. It has been demonstrated that electrolyte degradation takes place both on the carbon black surface and on the surface of the active material at high voltages.^29^ Furthermore, the surface area of carbon black has been found to be proportional to the extent of solvent oxidation and the degree of TM dissolution in the case of LiMnO_2_ cathodes.^30^ However, extracting information about the LNMO/electrolyte interface in itself is challenging based on the results from coated electrodes, as the coating is altering both the LNMO and the carbon black surface.

Kim et al.^31^ reported on ultrathin Al_2_O_3_ ALD coating (<1 nm) of commercial LNMO powder and found that the coating improved the Coulombic efficiency, cycle retention, and self-discharge behavior at 30 °C to some extent. The coating, however, also increased the overpotential and reduced the obtainable capacity. This corresponds well with the findings from Jung et al.,^32^ who compared Al_2_O_3_ coating of LiCoO_2_ (LCO) particles and LCO-containing electrode and found that coating of the active material by itself introduced a larger overpotential, which was attributed to the limited electronic conductivity in the Al_2_O_3_ film resulting from complete coverage of the LCO particles. Al_2_O_3_ coatings applied by solid-state sintering were found to offer only temporary protection from HF attack.^33,34^ This was attributed to the fact that Al_2_O_3_ is known to act as a HF scavenger, causing challenges with the consumption of the surface coating. Hall et al.^35^ showed in addition that Al_2_O_3_ can react directly with PF_6_^–^ in the electrolyte to form Al_2_O_3–*x*_F_2_ and AlF_3_. The fluorination of Al_2_O_3_ is accompanied by a substantial volume change, something that could induce the formation of cracks in the coating and exposure of bare, unprotected LNMO surface.^31^ Although improved electrochemical performance has been reported at 60 °C for Al_2_O_3_-coated NMC, for which the coating was applied by solution precipitation,^34^ an important aspect is the degree of protection by the Al_2_O_3_ coating over a prolonged time. While the current commercially available LNMO materials has demonstrated significantly improved cycling stability at room temperature over the last years (78% capacity retention has been achieved after 300 cycles^36^), the cycling stability drops dramatically at higher temperatures, which hinders the practical use of the materials for a number of applications. Thus, long-term protection from HF attack by the coating, and in particular at higher temperatures, is essential to justify the use of surface coatings. Further knowledge of the extent of protection over time, particularly at more extreme conditions such as elevated temperatures, is therefore needed to understand whether such coatings are beneficial to the battery lifetime.

In view of the scattered results previously reported on Al_2_O_3_ coatings, we have in this work investigated coatings applied by ALD, a technique that allows for accurate control of the coating thickness and the possibility to produce ultrathin and uniform surface coatings.^37^ Ultrathin, amorphous Al_2_O_3_ coatings were deposited on commercial LNMO and their influence on the electrochemical properties, cycling stability, and TM dissolution at higher temperatures was investigated. Three different thicknesses of Al_2_O_3_ coatings were deposited by exposing the LNMO to 5, 10, and 20 ALD cycles with trimethylaluminum (TMA) and H_2_O as precursors. The resulting materials were characterized by X-ray diffraction (XRD), Raman spectroscopy, scanning electron microscopy (SEM), inductively coupled plasma mass spectrometry, scanning transmission electron microscopy (STEM), and X-ray photoelectron spectroscopy (XPS) to investigate the quality of the coated materials. A systematic comparison of the electrochemical performance of the materials, with emphasis of cycling stability, was done at room temperature and at 50 °C, and TM dissolution was identified.

## Results and Discussion

### Materials Characterization

**Figure 1:**
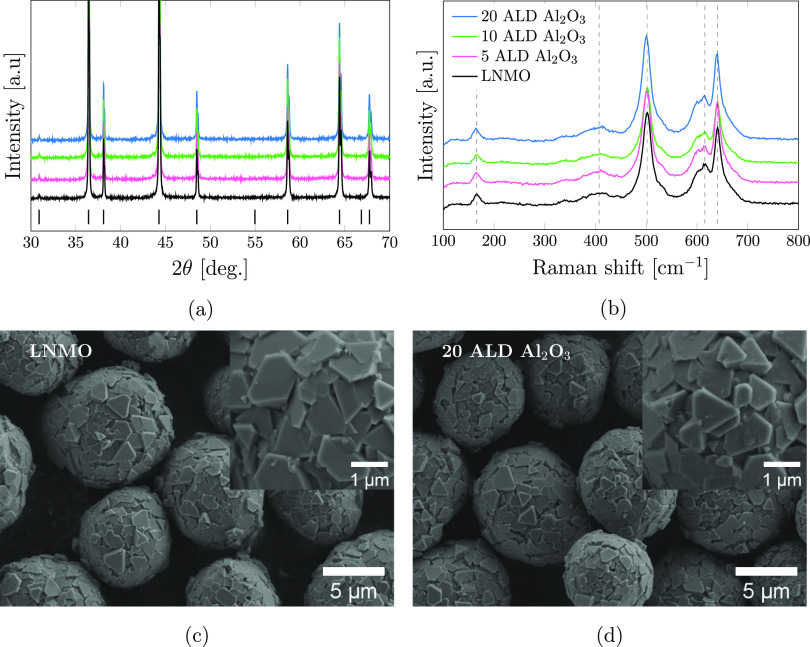
Powder XRD patterns (a) and Raman spectra (b) of pristine LNMO (black) 5 ALD Al_2_O_3_ (pink), 10 ALD Al_2_O_3_ (green), and 20 ALD Al_2_O_3_ (blue). The vertical bars in panel (a) indicate reflections for disordered LNMO with the space group *Fd*3̅*m* (PDF# 00-063-0626). The SEM micrographs show the morphology of the pristine LNMO powder (c) and the 20 ALD Al_2_O_3_ powder (d).

Three different thicknesses of Al_2_O_3_ were deposited on the LNMO powder by exposing it to 5, 10, and 20 ALD cycles. The samples are hereafter named as 5 ALD Al_2_O_3_, 10 ALD Al_2_O_3_, and 20 ALD Al_2_O_3_, respectively. Due to the low deposition temperature during the ALD coating procedure (120 °C), no change in the bulk LNMO is expected. Higher deposition temperatures and/or post-deposition heating steps are interesting approaches in order to change the crystallinity of the coating layer and increase the interfacial bonding strength between the LNMO substrate and the coating layer. Interdiffusion of the coating material into the bulk is, however, a possible additional effect, and careful parameter optimization in a separate study is required.^38^ From the X-ray diffractograms depicted in Figure 1a, no difference between the four LNMO powders is observed, and no introduction of new impurity phases in the coated samples is detectable. All samples display reflections, which can be indexed with the high-symmetry phase with the space group *Fd*3̅*m* of phase pure LNMO. This space group of LNMO is characterized by structural disorder where Mn and Ni cations are randomly positioned on the 16d sites and Li and O atoms are occupying 8a and 32e sites, respectively.^39^ The Raman spectra in Figure 1b confirm that the LNMO is predominantly disordered. The peaks at around 165 and 407 cm^–1^ correspond to Ni–O bands and are signatures of partial ordering in the spinel structure.^15,40,41^ The peak intensities of these features are expected to increase with increased ordering of the LNMO structure. The low observed intensity indicates that the degree of ordering in these samples is low. No peak shifts can be observed for the four samples; however, some small variations in peak intensity can be seen. Raman spectrometry, with a typical probing depth of 20–300 nm,^15^ is not surface sensitive enough to probe only the coating layer. These changes are thus not explained by the coating as most of the signal is coming from the LNMO bulk phase. These small variations can instead be assigned to the morphology of the particles. The morphology of the pristine LNMO and the 20 ALD Al_2_O_3_ powders are depicted in Figure 1c,d, respectively. The spherical secondary particles consist of polyhedral shaped primary particles resulting in a rough surface with many edges that can give rise to the observed intensity variations in the Raman spectra. There is no observable difference in the powder morphology of the pristine LNMO and the LNMO with the thickest Al_2_O_3_ coating (20 ALD Al_2_O_3_). Based on these results, we conclude that there is no apparent change in the bulk LNMO due to the coating procedure.

Inductively coupled plasma sector field mass spectrometry (ICP-SFMS) analysis was conducted on the uncoated and coated LNMO samples. A stoichiometry of Li_1.08_Ni_0.46_Mn_1.54_O_4_ is calculated from the measured average Li, Ni, and Mn content of all four samples, presented in Table S1, and corresponds well with the stoichiometry provided by the LNMO powder supplier (LiNi_0.43_Mn_1.57_O_4_). The measured Al content, presented in Table 1, is as expected increasing with increasing number of ALD cycles applied. The coating thickness for a homogeneous coverage was estimated by using the measured BET surface area of the LNMO powder (0.276 m^2^/g) and the density of the amorphous Al_2_O_3_ (from the work of Groner et al.^42^ estimated to be 2.7 g/cm^3^).

**Table 1:** Al Content as Measured by ICP-SFMS and the Calculated Coating Thickness for the Samples

sample name	Al content (RSD 15–25%) [mg/kg]	calculated coating thickness [nm]
LNMO	10	
5 ALD Al_2_O_3_	239	0.6
10 ALD Al_2_O_3_	373	1
20 ALD Al_2_O_3_	661	1.7

**Figure 2:**
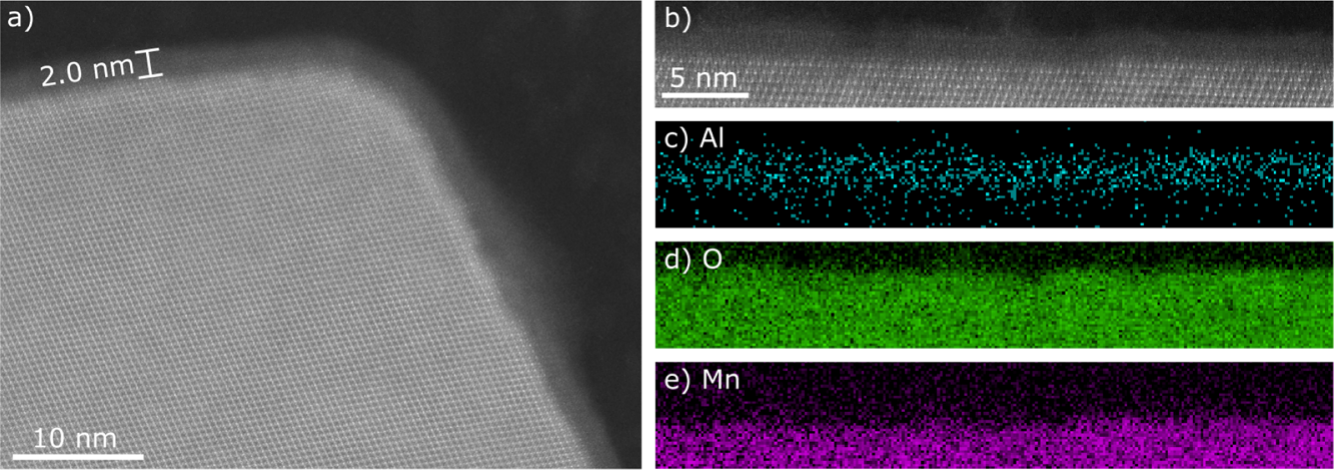
STEM results from the cross section of a 20 ALD Al_2_O_3_ particle. (a) ADF-STEM micrograph showing the uniformly thick coating over a facet corner. (b) Higher magnification ADF-STEM image of coating on a facet. (c–e) Corresponding EELS elemental maps from the Al–K, O–K, and Mn-L2,3 core loss edges, showing the presence of aluminum oxide.

The 20 ALD Al_2_O_3_ powder was analyzed by STEM coupled with electron energy loss spectroscopy (EELS). The uniformity and thickness of the coating layer are shown in Figure 2. Figure 2a shows an annular dark-field (ADF)-STEM micrograph. A uniform amorphous layer with an even thickness of approximately 2 nm is observed on the crystalline LNMO particle surface. The coating covers all the inner and outer corners between facets. The EELS elemental maps (Figure 2c–e) show that the surface layer contains Al and O. The observed coating thickness corresponds well with the findings of Cho et al.^43^ who estimated the Al_2_O_3_ coating thickness after 20 ALD cycles on LNMO electrodes to be slightly lower than 2 nm when applying the coating with TMA and H_2_O as precursors at 250 °C. The calculated coating thickness of 20 ALD Al_2_O_3_ (1.7 nm) based on the ICP-SFMS results also correspond well to the coating thickness observed in the STEM micrograph, further confirming the homogeneity of the coating.

**Figure 3:**
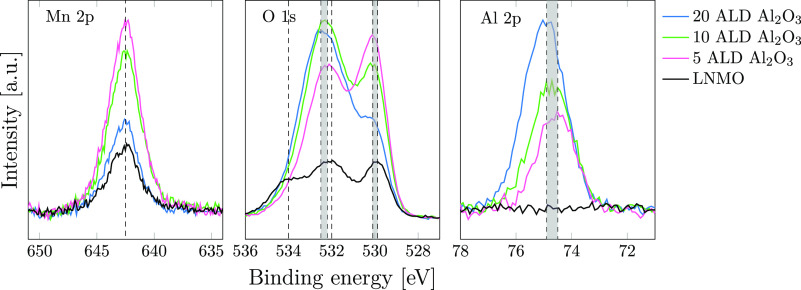
Mn 2p, O 1s, and Al 2p XPS spectra for the pristine LNMO (black), 5 ALD Al_2_O_3_ (pink), 10 ALD Al_2_O_3_ (green), and 20 ALD Al_2_O_3_ (blue).

The surface sensitivity of XPS makes it possible to probe the outermost surface of the particles, making this a suitable technique to gain additional information about the ultrathin Al_2_O_3_ coating. Electrodes containing the four samples, carbon black, and poly(vinylidene difluoride) (PVDF) were analyzed in XPS prior to electrochemical testing. The XPS elemental spectra (Mn 2p, O 1s, and Al 2p) of the electrodes are presented in Figure 3. In order to compare the samples, all the peak intensities have been normalized with respect to the F 1s peak and energy calibrated to C 1s at 285 eV. The F 1s peak was chosen as a reference as the F content from the PVDF binder is assumed to be comparable for all the samples. The uncoated LNMO (black) shows a lower intensity in the Mn 2p and O 1s spectra compared to the Al_2_O_3_-coated samples. The calculated surface elemental concentrations (in atomic percentages) included in the Supporting Information (Table S1) show that the measured carbon content in the pristine LNMO sample (81.6 at. %) is higher than for the Al_2_O_3_-coated samples (67.6–70 at. %), while the oxygen content is lower (3.5 at. % for uncoated LNMO and 8.5–9.1 at. % for the Al_2_O_3_-coated samples). This apparent difference in the C-content could be explained by the introduction of O on the surface by the Al_2_O_3_ coating, which will dilute the C concentration originating from the surface groups (such as C=O and C–O species) present on the pristine LNMO surface. The coating procedure could also change the affinity to accumulate surface-bound carbon due to the change in surface-bound groups. As the Al_2_O_3_ growth cycle is terminated with TMA exposure, the Al_2_O_3_-coated LNMO will, to a large extent, have −CH_3_-groups on the surface after ALD coating. Since the XPS analysis has been performed on electrodes, it cannot be excluded that the slurry process has affected the LNMO surface. The −CH_3_ surface groups on the Al_2_O_3_-coated samples could, as an example, react with moisture/air during the slurry process and form methanol, which in turn would leave the surface. By comparing the three Al_2_O_3_-coated powders, there is a clear trend with decreasing intensity in the Mn 2p peak with increasing Al_2_O_3_ coating thickness. This is expected as the increasing Al_2_O_3_ coating thickness will, to a larger extent, shield for the Mn 2p signal.

Three distinct peaks are present in the O 1s spectra for the uncoated LNMO, while two main peaks are present for the three Al_2_O_3_-coated samples. The peak at 529.9–530.1 eV, which is present in all spectra, is assigned to the metal oxide (M–O) (M = Ni, Mn, and Al).^44−46^ The peaks at 532 and 534 eV present in the uncoated LNMO spectrum are assigned to surface-bound C=O and C–O species, respectively.^46,47^ For the Al_2_O_3_-coated samples, the second peak at 532.2–532.5 eV is assigned to the metal hydroxide M–OH (M = Al).^44,45^ The presence of an Al–OH O 1s peak suggests that not all the Al–OH groups are replaced with Al–CH_3_ groups by the final TMA exposure in the coating procedure or that the slurry process has altered the surface-bound groups, as mentioned earlier. The relative intensity of the M–O O 1s peak to the M–OH O 1s peak is decreasing with increasing number of ALD cycles applied.

For the uncoated LNMO (black), there is as expected no Al 2p signal, while the Al 2p signal intensity is clearly increasing with increasing ALD coating thickness (5 ALD Al_2_O_3_ < 10 ALD Al_2_O_3_ < 20 ALD Al_2_O_3_). This supports the ICP-SFMS results where an increased Al amount was found for increasing number of ALD cycles applied. It furthermore indicates that Al is on the outermost surface of the LNMO particles and that the surface coating does not seem to be damaged to a large extent by the slurry and electrode coating process. There is a slight shift to higher BE values for the Al 2p peak with increasing coating thickness (from 74.5 eV for the 5 ALD Al_2_O_3_ to 74.9 eV for the 20 ALD Al_2_O_3_). This peak shift has been attributed to residual −OH groups at the outermost surface of the coated particles.^48^ It has also been suggested that amorphous structures will give rise to an Al 2p transition to a higher binding energy,^44^ and this shift can thus be an indication of increasing contributions from the amorphous Al_2_O_3_ phase with increasing coating thickness.

Taken together, the results show that the ALD coating strategy is successful, rendering homogeneous Al_2_O_3_ layers that uniformly cover the LNMO particles, and where the thickness follow the number of coating cycles. The analysis does not show any indications of contaminants in the coatings. Thereby, the coatings generated should be a useful platform for studying the influence on electrochemical performance.

### Electrochemical Characterization

**Figure 4:**
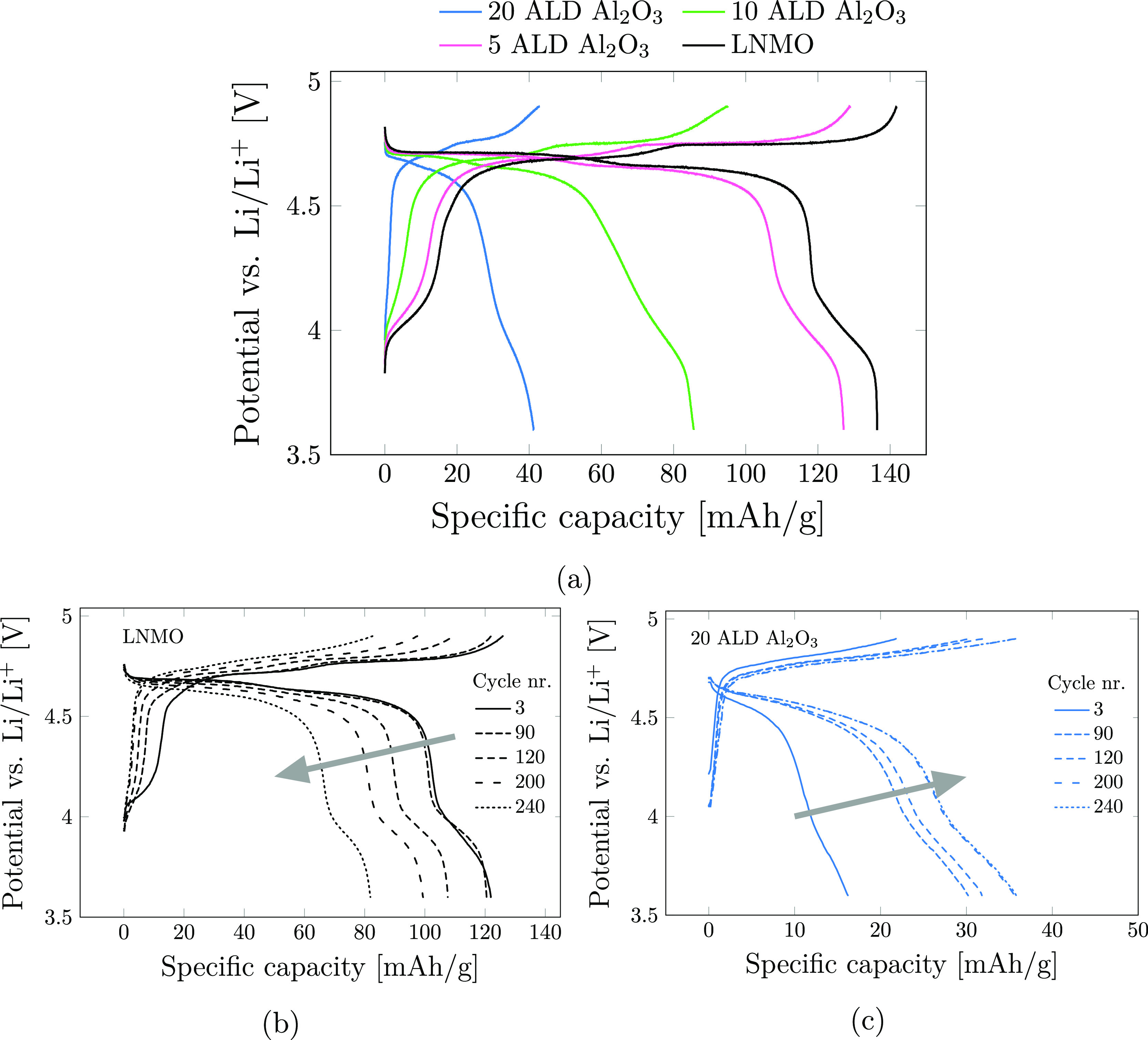
Second charge cycle at room temperature (a) for the pristine LNMO (black), 5 ALD Al_2_O_3_ (pink), 10 ALD Al_2_O_3_ (green), and 20 ALD Al_2_O_3_ (blue) at *C*/10. The charge–discharge curves showing the development with increasing number of cycles (indicated with arrows) for LNMO (b) and 20 ALD Al_2_O_3_ (c) with a current of *C*/2 for all plotted cycles.

The uncoated and coated LNMO powders were tested in CR-2032 coin cells with Li foil as a counter electrode. Cycling experiments were carried out both at room temperature and at 50 °C. The cells were initially cycled at *C*/10 between 3.6 and 4.9 V versus Li/Li^+^ for two cycles. Thereafter, the long-term cycling stability was evaluated at *C*/2 in the same potential range. The voltage curves of the second charging cycle at room temperature of all the four samples are shown in Figure 4a. The uncoated LNMO shows the typical voltage curve of disordered LNMO, with the two main plateaus around 4.7 V versus Li/Li^+^ (Ni^2+/4+^ redox activity) and a smaller plateau around 4 V versus Li/Li^+^ attributed to Mn^3+/4+^ redox activity.^15^ A clear effect of the Al_2_O_3_ coating on the reversible capacity and the polarization upon charge and discharge of LNMO is visible. The pristine LNMO shows the highest reversible capacity (136 mA h/g), while the obtained capacity is reduced slightly for the 5 ALD Al_2_O_3_ to 127 mA h/g. The 10 ALD cycle coated sample showed a reversible capacity of 85 mA h/g, and in the case of 20 ALD cycle coated sample, the capacity was reduced to merely 41 mA h/g. In contrast to several other studies where wet chemical,^49^ mechanochemical,^50^ and pulse laser deposition^51^ coating methods were applied, we found even a 2 nm thin Al_2_O_3_ coating layer to be severely detrimental to the initial reversible capacity. One possible reason for the variety in reported results is the differences in the degree of coating coverage. The Al_2_O_3_ coatings investigated in this study are very thin and uniform with continuous coverage. The ALD coatings have been deposited at 120 °C, most probably yielding a coating with a large degree of structural disorder. The Li-ion conductivity/diffusivity is expected to be very low for amorphous Al_2_O_3_ coatings.^26^ This, in addition to the band gap, which has been experimentally determined to be 9.9 eV [for thin (20 nm) amorphous films deposited onto silicon wafers],^52^ will make the Al_2_O_3_ coating a resistive layer that could act as a barrier for Li-ion diffusion and electronic conductivity. The complete coverage of the LNMO particles could thus be a disadvantage. A more inhomogeneous surface decoration on the other hand, which will result in more electrochemical active sites, could in fact be an advantage as it would not slow down the Li-ion diffusion to the same extent but still scavenge HF and in that way protect the active material from HF attack.

By looking at the development with increasing number of charge–discharge cycles (shown in Figure 4b,c for pristine LNMO and 20 ALD Al_2_O_3_, respectively), an opposite trend is observed for the pristine LNMO (black) and the 20 ALD Al_2_O_3_ (blue). The pristine LNMO shows increasing polarization and capacity decay with increasing number of cycles. This is expected due to decomposition products forming an interphase layer on the LNMO surface during cycling, leading to increased resistance for Li-ion diffusion. For the 20 ALD Al_2_O_3_, however, the trend is reversed. Increasing number of charging cycles results in a reduction in the polarization and increased capacity. Assuming that the Al_2_O_3_ coating is the main reason for the initial polarization, it is clear that the properties of the coating or the degree of coating coverage are changing during cycling. This is consistent with the findings of Kim et al.^31^ where this increase in capacity and reduced polarization with increasing number of charging cycles was assigned to the fluorination of Al_2_O_3_ that is accompanied by a volume change of 64%, which could lead to cracking of the surface coating and exposing of bare LNMO surface. The observed increase in discharge capacity with increasing number of charging cycles for the 20 ALD Al_2_O_3_ can alternatively be explained by a more gradual consumption of the Al_2_O_3_ coating through HF scavenging, leading to a thinner and less homogeneous coating layer that would allow for more facile Li-ion transport and consequently lower the polarization.

**Figure 5:**
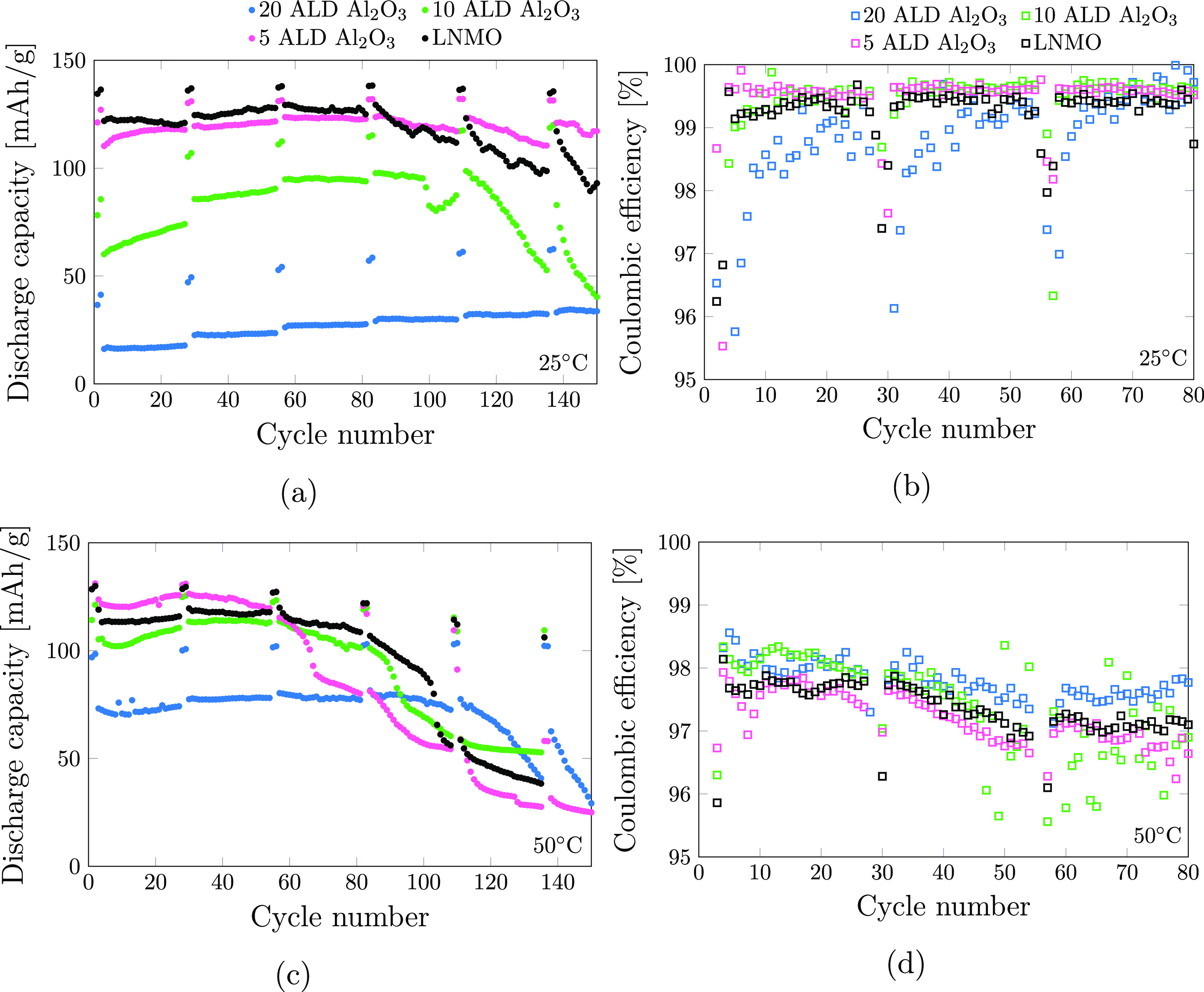
Discharge capacity and Coulombic efficiency for the cells cycled at room temperature (a,b) and the cells cycled at 50 °C (c,d) for pristine LNMO (black), 5 ALD Al_2_O_3_ (pink), 10 ALD Al_2_O_3_ (green), and 20 ALD Al_2_O_3_ (blue). The *C*-rates are *C*/2 for all cycles with two *C*/10 cycled every 25 cycles. Due to the large variation in cell behavior at 50 °C, the discharge capacity in (c) is the average value from 3 to 5 cells. The slow charging cycles (*C*/10) every 25th cycle is added to gain information about the origin of the capacity fade.

Long-term galvanostatic cycling, both at room temperature and at 50 °C, was performed. After two formation cycles at *C*/10, the cells were cycled at *C*/2 with two *C*/10 cycles every 25 cycles. Cycling at lower current rates can reveal whether the observed capacity decay originates from kinetic limitations or is caused by material degradation in Li excess systems such as Li metal half-cells. The discharge capacity for 150 cycles and the Coulombic efficiency at room temperature and at 50 °C are presented in Figure 5a–d. The high-temperature measurements presented in Figure 5c are average values from 3 to 5 cells as the onset of capacity decay varied. Only the first 150 cycles are presented for the measurements at 50 °C, as not all of the cells were cycled up to 270 cycles. Plots with standard deviations as error bars for the 50 °C measurements and all 270 cycles for the room temperature measurements are included in the Supporting Information (Figures S1 and S2, respectively). At room temperature, the uncoated and 5 ALD materials exhibit stable cycling up to 80 cycles at a capacity of 120 and 110 mA h/g, respectively. Afterward, capacity fading is observed at *C*/2, but the samples regained their initial capacity when cycled at *C*/10. The 10 and 20 ALD Al_2_O_3_ samples on the other hand show low initial capacities of 85 and 60 mA h/g, respectively. In contrast to the uncoated LNMO and 5 ALD Al_2_O_3_ samples, the 10 ALD and 20 ALD samples showed increasing capacity values at *C*/2 and *C*/10. The 10 ALD sample reached values of 100 mA h/g at *C*/10 and 120 mA h/g at *C*/2 after 120 cycles before this sample also began to lose capacity again. The 20 ALD showed a constant increase in capacity over 150 cycles, but even after 150 cycles, the capacity was still below 50 mA h/g. The total charge that passed through the material or the number of equivalent full cycles is hence lower and the time spent at high voltage is thus shorter for this sample, possibly resulting in reduced electrolyte decomposition and a more stable cycling. At 50 °C, a similar trend was observed, although the onset of capacity fade occurs after fewer charge–discharge cycles for all samples. For the first 60 cycles, the 5 ALD Al_2_O_3_ cycles at a higher capacity (120 mA h/g) than the uncoated LNMO (110 mA h/g), indicating a small improvement in the cycling properties at higher temperature for the 5 ALD Al_2_O_3_. The 10 and 20 ALD Al_2_O_3_ both show higher capacity at 50 °C than at room temperature before the capacity fade onset, with a capacity more similar to that of the uncoated LNMO. 10 ALD Al_2_O_3_ cycles at 100–110 mA h/g for *C*/2 and 120 mA h/g at *C*/10, while the 20 ALD Al_2_O_3_ cycles at 75 mA h/g at *C*/2 and 100 mA h/g at *C*/10. It has been found that the diffusivity of Li in Al_2_O_3_ obeys a near-ideal Arrhenius behavior,^26^ and the cycling performance is consistent with significantly improved Li^+^ transport properties through the Al_2_O_3_ coating at elevated temperatures. It should be noted that the extreme Li excess in half-cells can hide certain degradation effects, while the Li metal can introduce others. Björklund et al.^53^ showed that the cross talk between the Li metal anode and the NMC cathode leads to rapid capacity fading compared to when anode materials such as graphite and Li_4_Ti_5_O_12_ (LTO) were used. In addition, the carbon black constitutes a substantial part of the surface area of the electrode, even with just 5 wt % carbon black, and the electrolyte degradation occurs on the carbon black surface in addition to the surface of the active material at high voltages.^29^ The loss of capacity can thus be due to several undesired effects, but it can be assumed that the majority of the observed capacity fading is not due to degradation of the active material as all the cells regain most of their initial capacity in the cycles with a lower *C*-rate. This behavior shows that the capacity loss is most likely due to kinetic limitations from, for example, electrolyte degradation. To sum up, the results clearly show that the Al_2_O_3_ coating thicker than 0.6 nm has a detrimental effect on the electrochemical properties of LNMO. It furthermore does not improve the cycling stability in half cells at 50 °C. The 20 ALD Al_2_O_3_ shows stable cycling at room temperature, but the improved cycling stability comes with the cost of high overpotentials and a severely reduced reversible capacity.

The Coulombic efficiencies at room temperature, presented in Figure 5b, are quite similar for the uncoated LNMO, 5 ALD Al_2_O_3_, and 10 ALD Al_2_O_3_, with stable values above 99% for the first 80 cycles. Only the first 80 cycles are included in the figure, as the Coulombic efficiency values vary greatly after the capacity fade onset. The 20 ALD Al_2_O_3_ has a slightly lower Coulombic efficiency between 98 and 99%. All of the samples show lower Coulombic efficiencies for a few cycles right after the lower current rate cycles (of *C*/10). This effect is increasing with increasing Al_2_O_3_ coating thickness and is especially visible for the 20 ALD Al_2_O_3_ where the Coulombic efficiency drops down to 96% before it stabilizes above 98% after two to three additional charging cycles. The combined low Coulombic efficiency and increase in capacity during cycling for the 20 ALD Al_2_O_3_ electrode, and to a certain degree for the 10 ALD Al_2_O_3_, can indicate surface reactions that change the properties of the Al_2_O_3_ coating. At 50 °C, as presented in Figure 5d, all the samples have lower Coulombic efficiency than at room temperature, varying between 97 and 98.5%. This is not surprising, as the temperature instability of the LiPF_6_ salt^54^ and the increased instability of the LNMO∥Li system at higher temperature will lead to more unwanted side reactions for all samples and result in a lower Coulombic efficiency.

**Figure 6:**
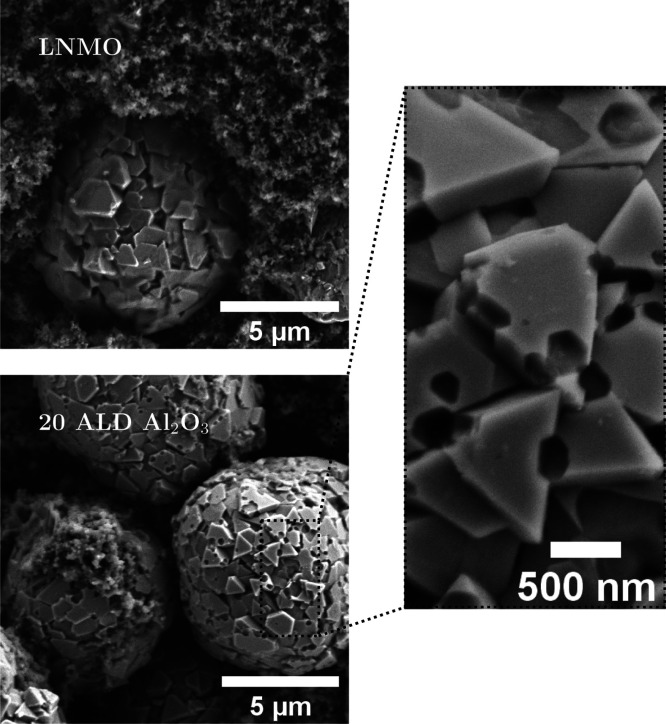
SEM micrographs showing LNMO (top) and 20 ALD Al_2_O_3_ (bottom) electrodes cycled at 50 °C for 270 cycles. Spots where the active material has been attacked are clearly visible on the 20 ALD Al_2_O_3_ particles.

As the TM dissolution is expected to increase with increasing temperature,^7^ the effect of Al_2_O_3_ coatings on the TM dissolution was investigated on cells cycled at 50 °C. Cycled LNMO cathodes were examined by SEM after 270 cycles at 50 °C. SEM micrographs are presented in Figure 6. No changes in the morphology could be discerned for the uncoated material. For the 20 ALD Al_2_O_3_, however, the primary particles have visible holes on the surface. The holes are relatively large (up to 100 nm in diameter) and extend into the active material. These holes are observed on several particles of the 20 ALD Al_2_O_3_ sample cycled at 50 °C. Neither of the electrodes cycled at room temperature had any LNMO particles with visible holes that could be observed with SEM. This also applies to the uncoated LNMO, 5 ALD Al_2_O_3_, and 10 ALD Al_2_O_3_ electrodes cycled at 50 °C. This does not exclude the presence of similar holes in these electrodes, but the extent of pitting formation is lower. The Al_2_O_3_ coating acts as a HF scavenger^33^ and is thus consumed via sacrificial reactions with HF.

HF can be generated by various routes in LiPF_6_-based carbonate electrolytes. The simple hydrolysis with trace amounts of water is well known and results in the formation of HF and POF_3_.^19,54,55^ Trace amounts of water can also react with EC forming ethylene glycol and CO_2_.^56^ The two-electron oxidation of ethylene glycol leads to the formation of glycolaldehyde and two protons, which will drive the dissociation of PF_6_^–^ into HF and PF_5_. The combination of high temperature and high potentials can cause yet another side reaction generating HF. Single-electron oxidation of EC forms an EC oxyradical species which will generate HF, CO_2_, and a reactive vinyl alkoxy radical in reaction with the PF_6_^–^ anion.^57^ The scavenging reactions of Al_2_O_3_ with HF generating aluminum fluoride were found by Myung et al.^34^ to take place stepwise according to eqs 1–3.
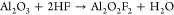
1
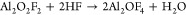
2
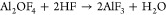
3

XPS analysis of pristine electrodes showed that Al–OH bonds are present at the surface of the Al_2_O_3_-coated LNMO samples. The presence of −OH groups on the particle surface may further increase the amount of water formed in the fluorization reaction. This is illustrated by looking at the total reaction of fluorination of Al_2_O_3_, presented in eq 4, and the fluorination of AlOOH, presented in eq 5.
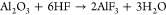
4
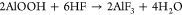
5

The sacrificial reaction of both Al_2_O_3_ and AlOOH will generate water where the presence of hydroxy group will result in the liberation of more water than the pure oxide. The formation of water can autocatalyze LiPF_6_ hydrolysis and hence further HF generation according to eq 6.^58^
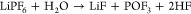
6

The catalytic cycle can thus lead to formation of new HF as the HF is consumed. A recent study by Tesfamhret et al.^59^ demonstrated that the amount of TM ions dissolved from the spinel LMO was higher for Al_2_O_3_-coated LMO than for pristine LMO. The increase in TM dissolution was attributed to the aforementioned catalytic HF/H_2_O cycle, and it can be assumed that Al_2_O_3_-coated LNMO will have similar issues. The very uniform Al_2_O_3_ coating layer generated by ALD could additionally lead to non-uniform conduction paths for Li, where Li ions only travel in and out of the particle at selected and electrochemically active points where the coating is thinner or absent, in particular for thicker Al_2_O_3_ coatings. This immense reduction in electrochemically active surface area could explain the high polarization and very low reversible capacity of the 20 ALD sample. In addition, these active sites could be more susceptible to HF attack and eventually lead to the formation of the observed holes in the LNMO particles, as they cause very localized water generation leading to further HF generation. The difference between our findings and several other published works on Al_2_O_3_-coated LNMO can thus be ascribed to the high degree of coating coverage on our Al_2_O_3_-coated LNMO particles which, surprisingly, leads to a disadvantage both in regard of electrochemical properties and in the degree of protection from HF attack.

**Figure 7:**
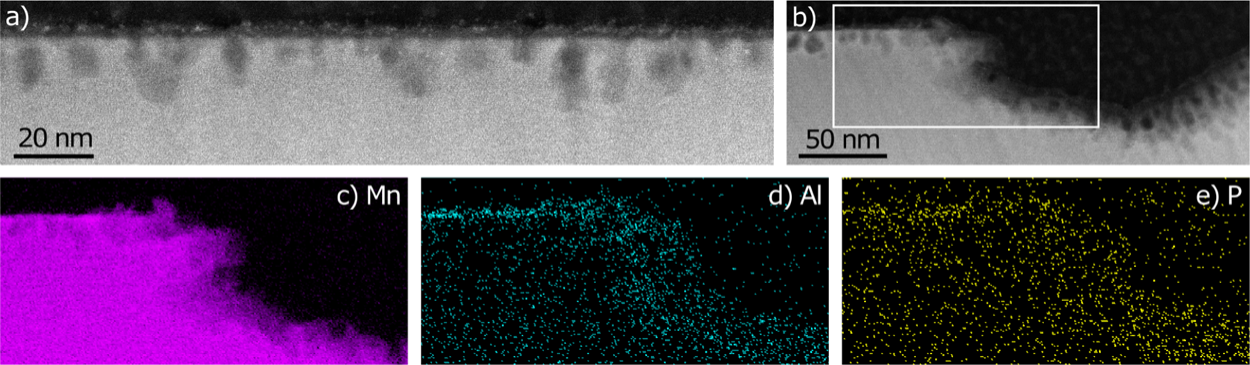
STEM results from the 20 ALD Al_2_O_3_ particle cross section. The particle is extracted from an electrode cycled at 50 °C for 270 cycles. (a) ADF-STEM image showing nano-sized holes in a faceted surface. (b) ADF-STEM image of a larger hole with corresponding (c) Mn EELS map, (d) Al EDX map, and (e) P EDX map. SEM and overview STEM images are available in the Supporting Information (Figure S3).

To further investigate the observed changes in the 20 ALD Al_2_O_3_ surface due to cycling at elevated temperatures, STEM analysis with EELS and energy-dispersive X-ray (EDX) spectroscopy mapping of a 20 ALD Al_2_O_3_ particle from an electrode cycled at 50 °C for 270 cycles was performed. The STEM image, depicted in Figure 7a, shows an overview of a larger faceted surface. Compared with the STEM image of the pristine 20 ALD Al_2_O_3_ particle in Figure 2, the particle surface is rougher after cycling. The even Al_2_O_3_ layer is no longer as distinct, and nano-sized holes that extend up to 20 nm into the LNMO particle are clearly visible. The holes are only a few nanometers apart and cover the majority of the particle surface. The crystal structure of LNMO is undisturbed in between the holes. This suggests that in addition to the larger holes, there is nucleation of similar sites along the whole particle surface as the material deteriorates. The ADF-STEM image depicted in Figure 7b shows the edge between the facet surface and a larger hole, similar to those visible in the lower SEM image in Figure 6. From the corresponding EELS and EDX maps (of Mn, Al, and P depicted in Figure 7c–e, respectively), it is clear that there is still Al present on the faceted surface after 270 charging cycles. As expected, there is a clear P signal from the surface, stemming from the LiPF_6_ salt and decomposed electrolyte products. F could not be detected due to the overlap between the F Kα and the Mn Lα peaks in EDX and a similar overlap problem in EELS, where the small F Kα peak will be hidden in the tail of the Mn Lα due to the low F concentrations. Overall, these results further confirm that the Al_2_O_3_ coating is not sufficiently protecting the LNMO surface after prolonged cycling at 50 °C.

**Figure 8:**
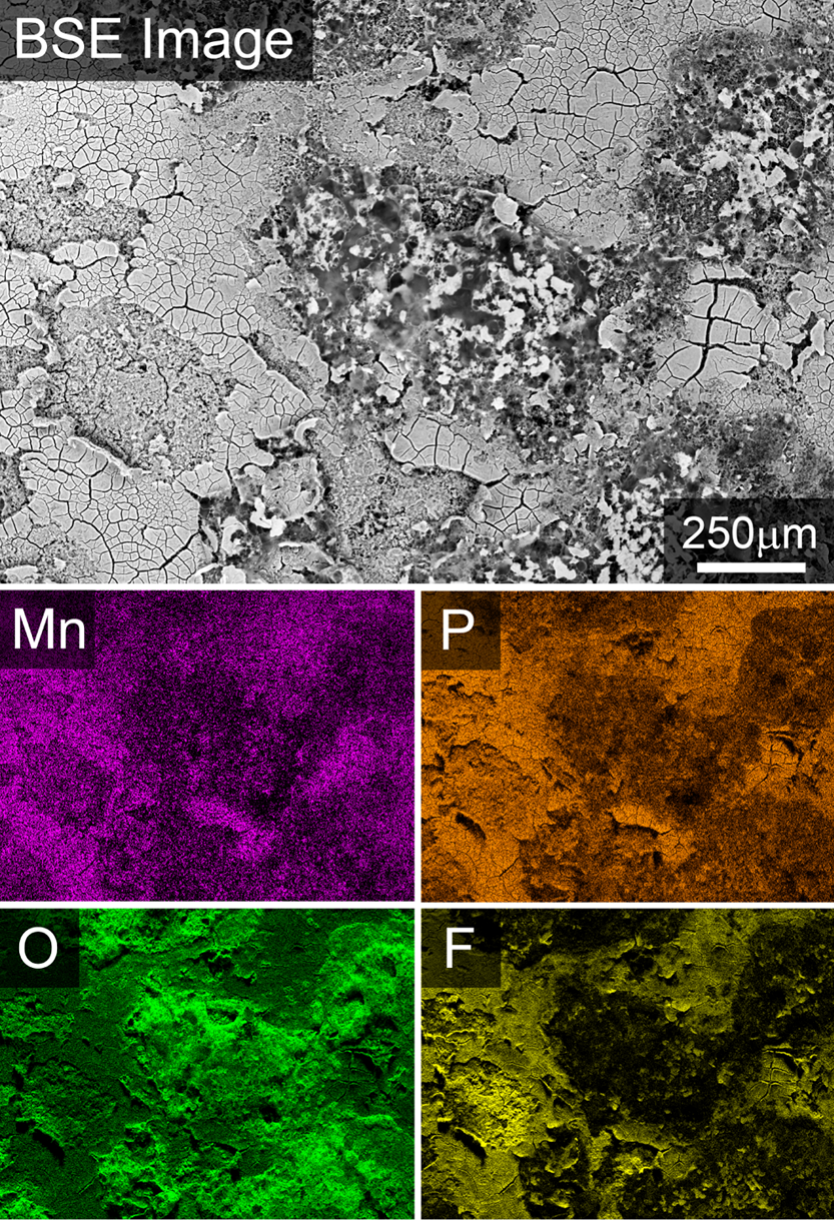
EDX image of a Li electrode cycled against 20 ALD Al_2_O_3_ at 50 °C for 270 cycles. A significant amount of Mn can be detected on all of the Li surface.

EDX analysis of the Li metal anode cycled against the 20 ALD Al_2_O_3_ at 50 °C for 270 cycles was performed and confirms that TM dissolution takes place in spite of the Al_2_O_3_ coating. The SEM micrograph and the EDX elemental maps are presented in Figure 8. The EDX spectra are included in the Supporting Information (Figure S4) and show that in addition to Mn, there are detectable amounts of Ni on the Li surface. In the SEM micrograph, the typical mossy structure of Li^60^ can be seen in the areas not covered by a thick SEI surface film. There is a detectable amount of Mn on the Li surface that seems to correlate well with the P and F signal in the SEI film. The O signal corresponds well with the mossy Li and is most likely caused by oxidation of Li during sample transfer in air. The presence of Mn and Ni is a clear indication that the Al_2_O_3_ coating does not prevent TM dissolution when cycled at higher temperatures. Even if most batteries are not going to operate at 50 °C, locally increased temperature in larger battery packs cannot be excluded and a similar behavior at room temperature after prolonged cycling can be suspected. The clear presence of Ni and Mn on the Li counter electrode in combination with the holes in the LNMO particles (for 20 ALD Al_2_O_3_ sample) gives an indication that uniform Al_2_O_3_ coating of a certain thickness (1.7 nm) could, due to the non-uniform current distribution, cause a more severe deterioration of the LNMO-active material than for the uncoated LNMO after prolonged cycling at 50 °C. Similar changes in micro- and nanostructure cannot be excluded for the 5 and 10 ALD Al_2_O_3_ samples, but the more homogeneous current distribution for these samples will presumably make this effect less prominent.

## Conclusions

In this study, the long-term protection by Al_2_O_3_ coating on LiNi_0.5–*x*_Mn_1.5+*x*_O_4−δ_ (LNMO) has been investigated. Ultrathin Al_2_O_3_ coatings were deposited on commercial LNMO powder by ALD. With a combination of characterization techniques, a uniform coating was verified, and the coating thickness was estimated to be approximately 1.7, 1, and 0.6 nm for the 20, 10, and 5 ALD cycles, respectively. Galvanostatic charge–discharge cycling in half-cells revealed that Al_2_O_3_ coating has a negative effect on the rate capability of LNMO, and there is little observed improvement in capacity retention for the Al_2_O_3_-coated LNMO compared to uncoated LNMO both at room temperature and at 50 °C in half-cells. The high overpotential observed for the Al_2_O_3_-coated samples in this study is attributed to the homogeneity of the deposited coating, leading to a Li-ion diffusion barrier. The homogeneous coating coverage may furthermore lead to non-uniform conduction paths for Li ions, forming active sites that are more susceptible for HF attack. As the coating is consumed, the overpotential is declining with increasing number of charging cycles, and pitting holes are observed in the 20 ALD Al_2_O_3_ LNMO particles after prolonged cycling at 50 °C. This, in addition to the clear presence of Ni and Mn on the Li surface after cycling, demonstrates that the use of ultrathin and uniform Al_2_O_3_ coatings will not inhibit TM ion dissolution from the high-voltage LNMO cathode over repeated cycling, particularly at higher temperatures.

## Experimental Section

LNMO with a chemical composition of LiNi_0.43_Mn_1.57_O_4_ was purchased from Haldor Topsøe (Denmark). Al_2_O_3_ powder coatings were deposited in a PICOSUN R-200 Standard ALD system at 120 °C to avoid condensation. TMA (EpiValence) and H_2_O were used as precursors. The precursor pulse time was set to 0.2 s at a flow rate of 15 sccm and a following carrier gas purge time of 5 s at a flow rate of 100 sccm. N_2_ was used as a carrier gas. The procedure was repeated 10 times to achieve a net of each precursor pulsing time of 2 s. The reactor was purged with carrier gas for 60 s at a flow rate of 600 sccm in between precursors. A subsequent exposure of the sample to the H_2_O and TMA precursors completed one growth cycle, and 5, 10, and 20 ALD growth cycles were implemented to prepare samples, named 5 ALD Al_2_O_3_, 10 ALD Al_2_O_3_, and 20 ALD Al_2_O_3_, respectively. The vacuum condition in the reaction chamber was controlled to under 10 hPa.

### Materials Characterization

X-ray diffractograms were recorded using a D8 Focus with Cu Kα radiation (λ = 1.54 Å) and LynxEye SuperSpeed Detector with a 6 h collection time over a 2θ range from 10 to 120°. Raman spectroscopy was performed with a Renishaw Raman spectrometer using 532 nm laser, 1200 grating, 50× lens magnification, and 0.5% laser power with a 20 s acquisition time. The lateral resolution is 1–2 μm and the probing depth is approximately 100 nm. SEM analysis of the LNMO particles was conducted on a Zeiss Ultra 55 limited edition field emission scanning electron microscope (FESEM), where the LNMO particles were connected to C-tape and analyzed with an acceleration voltage of 5 kV with a working distance of 5.5 mm and a 30 μm aperture. The BET surface area of the uncoated LNMO powder was measured using a TriStar 3000 surface area and porosity analyzer. The chemical composition of the samples was analyzed by ICP-SFMS using an ICP-SFMS Element 2 (Thermo Scientific, Bremen, Germany). For the sample preparation, coated and uncoated LNMO powders were dissolved in a mixture of hydrochloric acid, nitric acid, and hydrofluoric acid. The analysis was performed by ALS Scandinavia AB. All XPS analyses were performed using an Axis Ultra DLD X-ray photoelectron spectrometer with a monochromatic Al Kα X-ray source (10 mA, 10 kV). High-resolution regional maps were collected using 20 and 0.1 eV step size for each element. Preparations of the XPS samples were done in an Ar-filled glovebox (O_2_ and H_2_O levels <0.1 ppm), and the samples were transferred inert from the glovebox to XPS. All data analysis was performed using CasaXPS software, and Shirley background subtraction was used for data evaluation. STEM was done with a JEOL ARM-200F image- and probe-corrected microscope. A voltage of 200 kV and a beam current of 80 pA were used. The convergence angle was 27 mrad, and an annular dark-field detector with an inner collection angle of 35 mrad was used. EELS was performed using a GIF Quantum spectrometer with 35 mrad collection angle and 0.5 or 1 eV dispersion. Single-frame chemical maps were acquired with 10–40 ms dwell time. Preparation of TEM specimens was done using a Helios G4 dual-beam focused ion beam (FIB)-SEM instrument. LNMO particles were covered with electron-deposited and subsequently ion-deposited carbon. The particles were lifted out, attached individually to a Cu half-grid, and thinned to electron transparency with Ga ions. The final thinning was done with 2 kV ions.

### Electrochemical Characterization

Electrode coatings for all samples were produced by making a slurry consisting of 90 wt % LNMO, 5 wt % carbon black (Imerys C-NERGY SUPER C65), and 5 wt % Kynar Flex HFP 2801 PVDF dissolved in *N*-methyl-2-pyrrolidone solvent. To avoid damaging the Al_2_O_3_ coating and deagglomeration of the secondary LNMO particles, a gentle slurry mixing was performed using a RETSCH MM400 shaker mill with three ZrO_2_ balls (5 mm) at 25 Hz for 20 min. The slurry was coated onto 22 μm-thick carbon-coated Al foil (SDX, Showa Denko) with a gap size of 150 μm before they were dried overnight at 60 °C. Disc-shaped electrodes (12 mm) were cut and further densified at 21.7 MPa for 3 min using a uniaxial press. Before cell assembly, the electrodes were dried at 120 °C under dynamic vacuum for 12 h before transferring to an Ar-filled glovebox (O_2_ and H_2_O levels <0.1 ppm). The average LNMO loading was 5 mg/cm^2^. CR-2032 coin cells were assembled in an Ar-filled glovebox (O_2_ and H_2_O levels <0.1 ppm). A Celgard 2325 separator and Li foil (0.75 mm, Alfa Aesar) were used as the counter electrode. The electrolyte (40 μL, 1 M LiPF_6_ in 1:1 EC/DEC from Alfa Aesar) was added by a micropipette. Galvanostatic cycling was conducted using a LAND battery testing system (CT2001A) both at room temperature and at 50 °C with *C*-rates of 0.5 C with two charge–discharge cycles of 0.1 C every 25th charge cycle. 1 C corresponds to a current of 140 mA h/g. Preparations of the SEM specimens for post-mortem analysis were performed by opening the cycled cells in an Ar-filled glovebox where the cathodes were extracted. They were then left to dry before they were removed from the glovebox in a sealed, Ar-filled container. The electrodes were exposed to air for a maximum of 30 s during sample transfer. A Zeiss Ultra 55 limited edition FESEM was used for the analysis, with a voltage of 5 kV with a working distance of 5.5 mm and a 30 μm aperture. To avoid any Mn contamination from the steel casing of coin cells for the EDX post-mortem analysis, pouch cells were prepared to cycle Li metal against the 20 ALD Al_2_O_3_ cathode at high temperatures. A double-separator layer (Celgard 2325) was used to ensure no direct contact between the 20 ALD Al_2_O_3_ and the Li metal. The pouch cells were opened in an Ar-filled glovebox (O_2_ and H_2_O levels <0.1 ppm) and transported in a sealed container. The Li metal anodes were exposed to air for 5 min during sample transfer. The analysis was conducted with a Hitachi S-3400N SEM.

## Supporting Information Available

Additional ICP-MS data; calculated atomic percentages; long-term cycling data; SEM image of 20 ALD Al_2_O_3_ particle selected for FIB cross-sectioning; and elemental spectra of Li counter electrode (PDF)
