# Supplementary material for: Limitations of Ultrathin Al2O3 Coatings on LNMO Cathodes
Source: ACS Omega. 2021 Nov 3;6(45):30644–55. doi: 10.1021/acsomega.1c04457 (PMC8603187; doi:10.1021/acsomega.1c04457)
Supplement: Supplementary file 1 — ao1c04457_si_001.pdf [file ao1c04457_si_001.pdf]

Limitations of ultra-thin Al_2O_3-coatings on LNMO cathodes

Elise R. Østli,[*,†] Yonas Tesfamhret,[‡] Sigurd Wenner,[¶] Matthew J. Lacey,[§] Daniel Brandell,[‡] Ann Mari Svensson,[†] Sverre M. Selbach,[†] and Nils P. Wagner[*,†,¶]

†*Department of Materials Science and Engineering, NTNU Norwegian University of Science and Technology, 7491 Trondheim, Norway*

‡*Department of Chemistry - Ångström Laboratory, Uppsala University, Box 538, 75121 Uppsala, Sweden*

¶*Sintef Industry, 7491 Trondheim, Norway*

§*Scania CV AB, 151 32 Södertälje, Sweden*

E-mail: elise.r.ostli@ntnu.no; nils.p.wagner@ntnu.no

Supporting Information

Inductively coupled plasma mass spectrometry (ICP-MS) was executed to gain information about the Al amount of the Al_2O_3-coated samples, as well as the stoichiometry of the LNMO powder. The measured amounts of Li, Ni, and Mn for all powder samples (LNMO, 5 ALD Al_2O_3, 10 ALD Al_2O_3, and 20 ALD Al_2O_3) in addition to the calculated average values are presented in Table S1. The stoichiometry calculated based on the average Li, Ni, and Mn contents is $Li_{1.08}Ni_{0.46}Mn_{1.54}O_4$. This corresponds well with the stoichiometry of $LiNi_{0.43}Mn_{1.57}O_4$ provided by the supplier.

Table S1: Li, Mn, and Ni amounts in LNMO, 5 ALD Al_2O_3, 10 ALD Al_2O_3, and 20 ALD Al_2O_3 powder samples as measured by ICP-MS.

Sample name	Li* [mg/kg]	Mn* [mg/kg]	Ni* [mg/kg]
LNMO	5.78	8.15	2.36
5 ALD Al_2O_3	5.82	8.34	2.36
10 ALD Al_2O_3	5.76	8.39	2.51
20 ALD Al_2O_3	5.78	8.17	2.42
Average	5.78	8.26	2.43

*RSD 15-25%

The calculated surface elemental concentration of Al, C, F, Mn, and O in the pristine electrodes containing LNMO, 5 ALD Al_2O_3, 10 ALD Al_2O_3, and 20 ALD Al_2O_3 based on X-ray photoelectron spectroscopy (XPS) measurements is presented in Table S2.

Table S2: Calculated atomic percentage of elements in pristine electrodes containing LNMO, 5 ALD Al_2O_3, 10 ALD Al_2O_3, and 20 ALD Al_2O_3 obtained from XPS.

Sample name	Al [At%]	C [At%]	F [At%]	Mn [At%]	O [At%]
LNMO	0	81.6	14.6	0.2	3.6
5 ALD Al_2O_3	4.9	70.1	15.8	0.7	8.5
10 ALD Al_2O_3	5.9	68.6	15.4	0.6	9.5
20 ALD Al_2O_3	6.1	67.6	16.9	0.3	9.1

The discharge capacity for 150 cycles at 50°C is presented in Figure S1. The presented data is average values from 3-5 cells, included standard deviations as error bars, as the onset

of capacity decay varied between the cells.

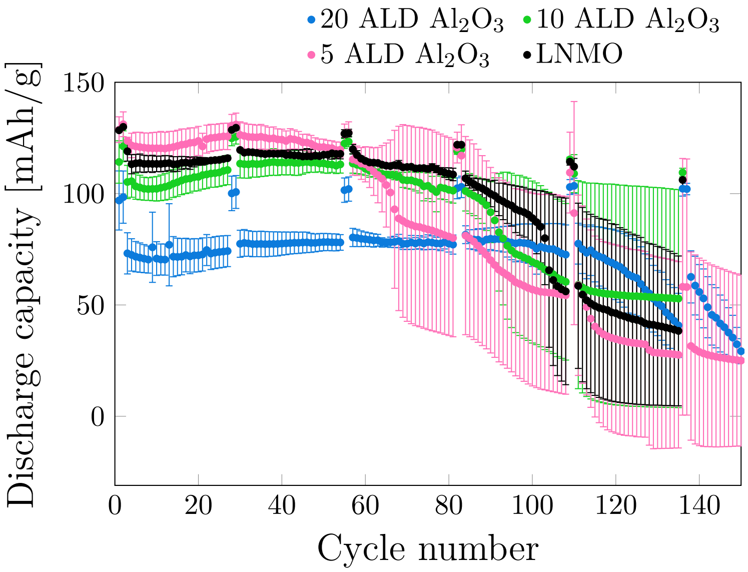

Figure S1: Discharge capacity with error bars for the cells cycled at 50°C for pristine LNMO (black), 5 ALD Al_2O_3 (pink), 10 ALD Al_2O_3 (green), and 20 ALD Al_2O_3 (blue). The C-rates are C/2 for all cycles with two C/10 cycled every 25 cycles.

The discharge capacity for all 270 cycles at room temperature is presented in Figure S2.

The particle selected for FIB cross-sectioning for the STEM analysis of the 20 ALD Al_2O_3 particle from an electrode cycled at 50°C for 270 cycles is presented in Figure S3. Several of the holes that are visible in the SEM image are included in the cross-section (marked by red circles). The areas scanned for the STEM image and EDX and EELS maps included in the results section is marked in the lower STEM image.

The backscattered electron (BSE) image of Li electrodes cycled against 20 ALD Al_2O_3 at 50°C for 270 cycles including elemental spectra is presented in Figure S4. Mn and Ni can be detected both in area 1 (surface SEI film) and area 2 (mossy Li surface), but it is more prominent on the parts of the electrode that is covered by the SEI film.

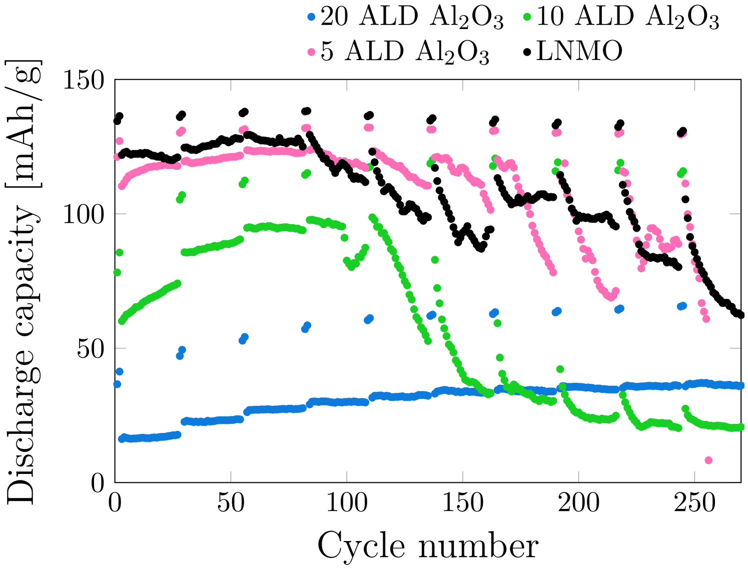

Figure S2: Discharge capacity for the cells cycled at room temperature for pristine LNMO (black), 5 ALD Al_2O_3 (pink), 10 ALD Al_2O_3 (green), and 20 ALD Al_2O_3 (blue). The C-rates are C/2 for all cycles with two C/10 cycled every 25 cycles.

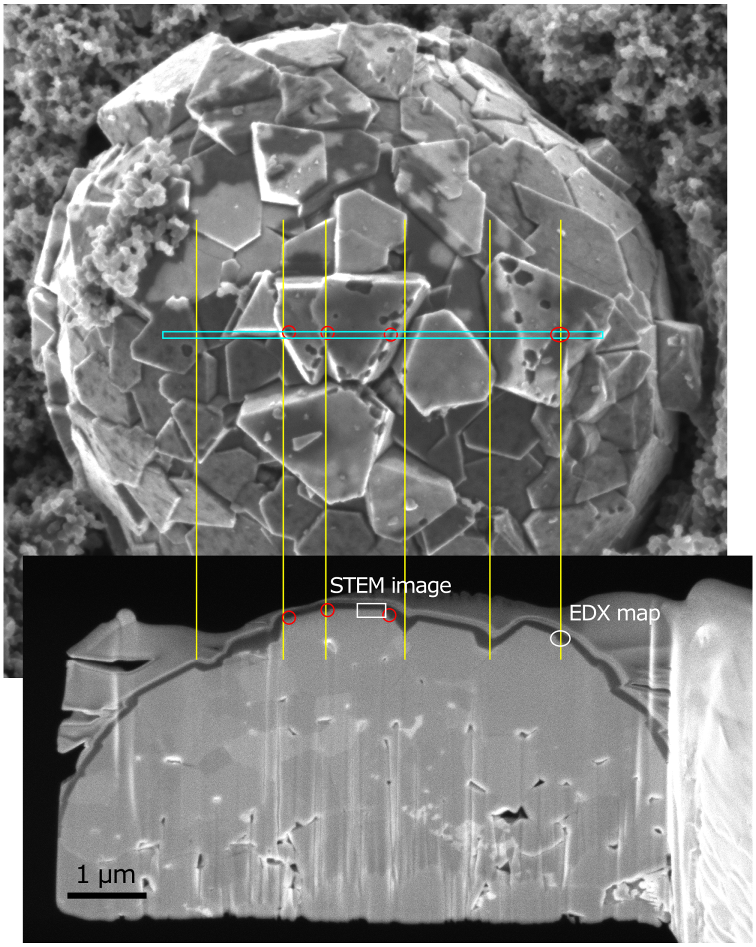

Figure S3: (upper) SEM image of 20 ALD Al_2O_3 particle selected for FIB cross-sectioning. (lower) STEM image of the cross-section, with landmarks and holes marked corresponding to the SEM image.

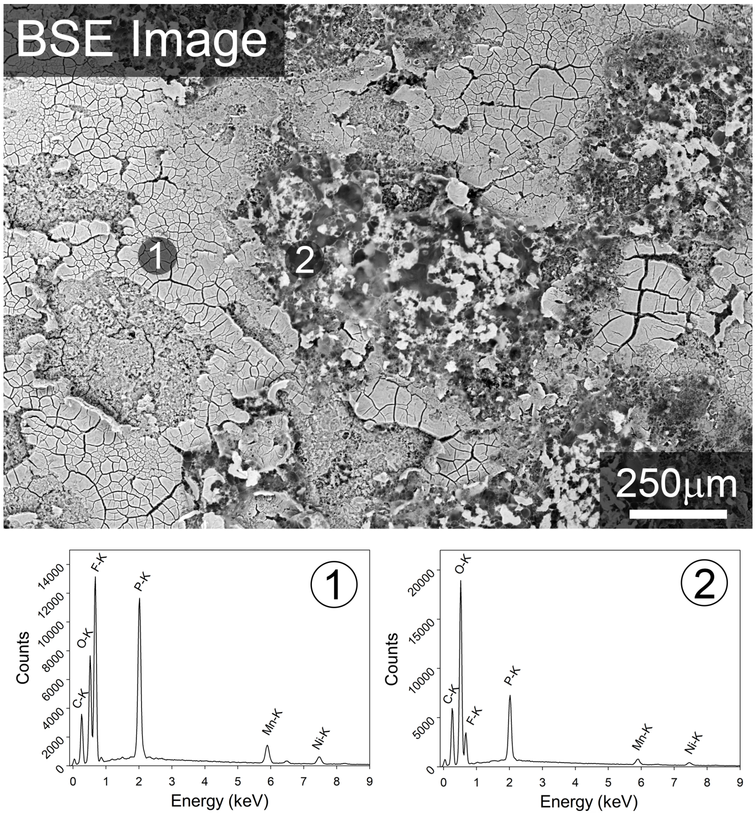

Figure S4: BSE image of Li electrodes cycled against 20 ALD Al_2O_3 at 50°C for 270 cycles. Elemental spectra show the presence of both Mn and Ni in both area 1 and 2.
